# Optimal timing of initiating continuous renal replacement therapy in septic shock patients with acute kidney injury

**DOI:** 10.1038/s41598-019-48418-4

**Published:** 2019-08-19

**Authors:** Bo Ra Yoon, Ah Young Leem, Moo Suk Park, Young Sam Kim, Kyung Soo Chung

**Affiliations:** Division of Pulmonology, Department of Internal Medicine, Yonsei University College of Medicine, Seoul, South Korea

**Keywords:** Bacterial infection, Continuous renal replacement therapy, Acute kidney injury

## Abstract

Acute kidney injury (AKI) in patients with septic shock is associated with high mortality, but the appropriate timing for initiating continuous renal replacement therapy (CRRT) is controversial. We retrospectively enrolled 158 septic shock patients with AKI in the medical intensive care unit (ICU) from July 2016 to April 2018. The time from AKI onset to CRRT initiation was compared according to ICU mortality using Cox proportional hazard, receiver operating characteristic, and Kaplan-Meier survival analyses. At the time of ICU discharge, the mortality rate was 50.6% (n = 80). It took longer to initiate CRRT in non-survivors than in survivors (hazard ratio 1.009; 95% confidence interval [CI] 1.003–1.014; *P* = 0.002). The cut-off time from AKI onset to CRRT initiation for ICU mortality was 16.5 hours (area under the curve 0.786; 95% CI 0.716–0.856; *P* < 0.001). The cumulative mortality rate was significantly higher in patients in whom CRRT was initiated beyond 16.5 hours after AKI onset than in those in whom CCRT was initiated within 16.5 hours (log-rank test, *P* < 0.001). Several clinical situations must be considered to determine the optimal timing of CRRT initiation in these patients. Close observation and CRRT initiation within 16.5 hours after AKI onset may help improve survival.

## Introduction

In critically ill patients, septic shock is the cause of acute kidney injury (AKI) in more than 40% of patients in the intensive care unit (ICU). About 70% of patients with AKI require renal replacement therapy (RRT), and in-hospital mortality is more than 60%^1,2^.

RRT is known to improve the ICU survival rate because it corrects metabolic acidosis by regulating lactate removal, unmeasured anions, and levels of phosphate and chloride^3,4^. In the patients with septic shock, RRT performs other roles in addition to the conventional renal function replacing. Modulating the immune response and maintaining hemodynamic stability in septic shock patients have an impact on the survival rate, and it is known to be more effective in continuous RRT (CRRT) than in intermittent haemodialysis (IHD)^5–8^. However, considering the potential complications associated with RRT, the optimal timing of initiating CRRT in septic shock patients with AKI remains controversial.

Most previous prospective, randomised controlled trials about the CRRT initiation time were performed on general, non-surgical ICU patients. However septic shock is usually life-threatening compared to other clinical conditions observed in the ICU, it is difficult to apply the results of these studies to septic shock patients that conducted on the patients with various degrees of severity. As the same conditions, most studies on patients with septic shock have been performed retrospectively, and the few prospective studies that have been conducted were limited to patients with a low severity.

According to the Kidney Disease: Improving Global Outcomes (KDIGO) Clinical Practice Guideline for AKI, the definition of AKI is clear. The initiation time of RRT, however, is referred to as ‘when life-threatening changes in fluid, electrolyte, and acid-base balance exist’; moreover, when we initiate RRT in clinical practice, it is recommended to consider ‘conditions that can be modified with RRT and trends of laboratory tests, rather than single BUN [blood urea nitrogen] and creatinine thresholds alone’^9^. Therefore, the concept of ‘optimal timing’ in CRRT initiation has no clear consensus, and the terms ‘early’ and ‘late’ CRRT initiation are widely defined in previous studies; the development of clinical manifestations and complications of renal insufficiency, requirement of inotropic drugs, and blood urea nitrogen (BUN) levels are indiscriminate in some patients^10–15^.

We began our research with the following practical and basic questions. How do the time from AKI onset to CRRT initiation influence on the survival of septic shock patients? When is the optimal timing to initiate CRRT in septic shock patients with AKI? Therefore, in this study, we analysed the relationship between the period from AKI onset to CRRT initiation and ICU mortality and attempted to suggest an appropriate timing of CRRT to increase the survival rate in septic shock patients with AKI.

## Results

### Baseline characteristics of the study population

One hundred fifty-eight patients were enrolled in this study, and the ICU mortality rate was 50.6% (80 patients). The baseline demographics at the time of admission to the ICU are presented in Table 1. Compared to survivors, non-survivors had a higher SOFA score (survivors versus [vs.] non-survivors; 10 vs. 12, *P* = 0.007), Acute Physiology and Chronic Health Evaluation Score II (APACHE II) (25 vs. 28; *P* = 0.029), and Simplified Acute Physiology Score II (SAPS II) (47 vs. 54, *P* = 0.001). There was no statistically significant difference in age, sex, body mass index, and comorbid diseases between the groups. There was also no statistically significant difference in the source of infection between the groups (*P* = 0.207). However, urinary tract infection (17 patients [21.8%] vs. 5 patients [6.3%]) was more common in survivors, while respiratory tract infection (26 patients [33.3%] vs. 41 patients [51.2%]) was more frequent in non-survivors. Compared to survivors, non-survivors had a lower mean arterial pressure (MAP) (73 mmHg vs. 63 mmHg, *P* = 0.018), lower albumin level (2.5 g/dL vs. 2.3 g/dL, *P* = 0.004), and higher total bilirubin level (1.0 mg/dL vs. 1.9 mg/dL, *P* = 0.034). However, the white blood cell (WBC) count (16 × 10^3^/µL vs. 13 × 10^3^/µL, *P* = 0.031) and procalcitonin level (117.65 ng/mL vs. 5.10 ng/mL, *P* = 0.040) were higher in survivors than in non-survivors. There was a statistically significant difference between survivors and non-survivors in the sodium levels (138 mmol/L vs. 141 mmol/L, *P* = 0.037), but they were within normal range (reference, 135–145 mmol/L).

**Table 1:** Baseline characteristics for septic shock patients who admitted to ICU because of continuous renal replacement therapy.

	Survivors n = 78 (49.4%)	Non-survivors n = 80 (50.6%)	Total n = 158	*P value*
Age (year)	69 (19, 94)	66 (25, 93)	68 (19, 94)	*0*.*185*
Sex (male), n (%)	50 (64.1)	47 (58.8)	97 (61.4)	*0*.*491*
BMI (kg/m^2^)	22.6 (19.9, 27.3)	23.0 (21.0, 26.1)	22.9 (20.1, 26.5)	*0*.*731*
SOFA score	10 (8, 12)	12 (9, 14)	11 (9, 13)	*0*.*007*
APACHE II score	25 (19, 33)	28 (22, 37)	27 (20, 35)	*0*.*029*
SAPS II score	47 (37, 55)	54 (43, 66)	50 (40, 61)	*0*.*001*
CCI	3 (2, 5)	4 (1, 5)	3 (2, 5)	*0*.*260*
Comorbidity disease, n (%)				
Congestive heart failure	3 (3.8)	7 (8.8)	10 (6.3)	*0*.*207*
Chronic pulmonary disease	7 (9.0)	8 (10.0)	15 (9.5)	*0*.*827*
CKD^¥^	21 (26.9)	14 (17.5)	35 (22.2)	*0*.*155*
Solid cancer	24 (30.8)	30 (37.5)	54 (34.2)	*0*.*374*
Hematologic malignancy	4 (5.1)	9 (11.3)	13 (8.2)	*0*.*163*
Mechanical ventilation used, n (%)	38 (48.7)	67 (83.8)	105 (66.5)	<*0*.*001*
Source of infection, n (%)				*0*.*302*
Gastrointestinal	29 (37.2)	29 (36.3)	58 (36.7)	
Respiratory	26 (33.3)	41 (51.2)	67 (42.4)	
Urinary tract	17 (21.8)	5 (6.3)	22 (13.9)	
Soft tissue	5 (6.4)	2 (2.5)	7 (4.4)	
Central nervous system	1 (1.3)	0 (0.0)	1 (0.6)	
Miscellaneous	0 (0.0)	3 (3.8)	3 (1.9)	
Clinical parameters				
Mean arterial pressure (mmHg)	73 (59, 90)	63 (53, 77)	68 (55, 86)	*0*.*018*
Heart rate (beats/min)	113 (95, 127)	116 (94, 129)	114 (95, 128)	*0*.*529*
WBC (x10^3^/μL)	16 (10, 22)	13 (5, 21)	14.6 (9.3, 22.2)	*0*.*031*
Platelet (x10^3^/μL)	93 (46, 193)	74 (37, 151)	83 (42, 175)	*0*.*115*
Albumin (g/dL)	2.5 (2.2, 2.8)	2.3 (1.8, 2.6)	2.4 (2.0, 2.7)	*0*.*004*
T.bilirubin (mg/dL)	1.0 (0.5, 2.5)	1.9 (0.7, 3.6)	1.2 (0.6, 3.3)	*0*.*034*
BUN, (mg/dL)	44.0 (31.2, 65.7)	44.5 (31.9, 63.7)	44.0 (31.8, 64.8)	*0*.*836*
Creatinine (mg/dL)	2.44 (1.45, 3.34)	2.34 (1.55, 3.07)	2.43 (1.51, 3.10)	*0*.*766*
Sodium (mmol/L)	138 (134, 145)	141 (138, 146)	140 (135, 145)	*0*.*037*
Potassium (mmol/L)	4.3 (3.6, 5.1)	4.3 (3.7, 5.0)	4.3 (3.7, 5.0)	*1*.*000*
Lactate, (mmol/L)	3.4 (2.2, 5.6)	9.7 (4.4, 15.3)	5.0 (2.6, 11.7)	<*0*.*001*
Urine output, (ml/day)	585 (196, 1,381)	520 (188, 1,070)	570 (189, 1,206)	*0*.*321*
Procalcitonin (ng/mL)	17.65 (2.77, 63.81)	5.10 (1.23, 34.35)	7.89 (1.44, 38.76)	*0*.*040*
CRP (mg/L)	162.6 (42.6, 269.4)	116.9 (62.1, 202.0)	128.8 (51.9, 226.2)	*0*.*310*

Values are expressed as n(%) or median(interquartile range) unless otherwise indicated; ^¥^Of the CKD patients GFR ≥ 15 ml/min/1.73 m^2^ were included.

BMI, Body Mass Index; SOFA, Sequential Organ Failure Assessment; APACHE II, Acute Physiology and Chronic Health Evaluation Score II; SAPS II, Simplified Acute Physiology Score II; CCI, Charlson Comorbidity Index; CKD, Chronic Kidney Disease; WBC, White Blood Cell; BUN, Blood Urea Nitrogen; CRP, C-Reactive Protein.

### Comparison of renal function

A comparison of the renal function parameters at the time of AKI occurrence, ICU admission, and initiation of CRRT is shown in Table 2. At the time of AKI onset, the BUN level (50.0 mg/dL vs. 40.5 mg/dL, *P* = 0.047), serum creatinine (SCr) level (2.54 mg/dL vs. 1.88 mg/dL, *P* = 0.003), and potassium level (4.7 mmol/L vs. 4.4 mmol/L, *P* = 0.024) were higher in survivors than in non-survivors. The estimated glomerular filtration rate (eGFR) (24 mL/min/1.73 m^2^ vs. 31 mL/min/1.73 m^2^, *P* = 0.020) was lower in survivors than in non-survivors. However, the aforementioned parameters did not show a statistically difference at ICU admission and CRRT initiation. The lactate level was not statistically significant at the time of AKI occurrence, but compared with survivors it was higher in non-survivors at ICU admission (survivors versus [vs.] non-survivors; 3.4 mmol/L vs. 9.7 mmol/L, *P* < 0.001) and CRRT initiation (4.3 mmol/L vs. 7.5 mmol/L, *P* = 0.001). The pH measured in arterial blood gas analysis was significantly lower in non-survivors than in survivors only at the time of ICU admission (survivors versus [vs.] non-survivors; 7.379 vs. 7.300, *P* < 0.001).

**Table 2:** Renal function parameters of baseline, at the time of AKI occurrence and CRRT initiation.

	Survivors n = 78 (49.4%)	Non-survivors n = 80 (50.6%)	*P* value
At acute kidney injury occurrence			
Lactate (mmol/L)	4.9 (2.8, 9.8)	7.0 (3.7, 12.6)	*0*.*113*
BUN (mg/dL)	50.0 (30.5, 70.6)	40.5 (26.2, 57.5)	*0*.*047*
Creatinine (mg/dL)	2.54 (1.54, 3.66)	1.88 (1.27, 2.88)	*0*.*003*
Potassium (mmol/L)	4.7 (1.0, 5.8)	4.4 (3.7, 5.1)	*0*.*024*
Urine output (ml/day)	1,493 (505, 2,140)	1,114 (467, 2,487)	*0*.*698*
pH	7.295 (7.236, 7.389)	7.299 (7.209, 7.395)	*0*.*963*
GFR (ml/min/1.73 m^2¥^)	24 (16, 39)	31 (19, 53)	*0*.*020*
GFR (ml/min/1.73 m^2€^)	23 (15, 42)	32 (18, 57)	*0*.*015*
At ICU admission			
Lactate (mmol/L)	3.4 (2.2, 5.6)	9.7 (4.4, 15.3)	<*0*.*001*
BUN (mg/dL)	44.0 (31.2, 65.7)	44.5 (31.9, 63.7)	*0*.*836*
Creatinine (mg/dL)	2.44 (1.45, 3.34)	2.34 (1.55, 3.07)	*0*.*766*
Potassium (mmol/L)	4.3 (3.6, 5.1)	4.3 (3.7, 5.0)	*1*.*000*
Urine output (ml/day)	585 (196, 1,381)	520 (188, 1,070)	*0*.*321*
pH	7.379 (7.306, 7.430)	7.300 (7.251, 7.374)	<*0*.*001*
At CRRT initiation			
Lactate (mmol/L)	4.3 (2.6, 8.9)	7.5 (4.2, 15.5)	*0*.*001*
NGAL (ng/mL)	1,004 (465, 1,695)	820 (367, 2,083)	*0*.*488*
Cystatin C (mg/L)	2.76 (1.87, 3.89)	2.89 (2.04, 3.93)	*0*.*640*
GFR (ml/min/1.73 m^2≠^)	14 (8, 28)	14 (8, 25)	*0*.*829*
Interval time from AKI to CRRT initiation (hours)	9 (6, 14)	26 (11, 66)	<*0*.*001*
CRRT duration (hours)	78 (52, 146)	67 (31, 212)	*0*.*607*

Values are expressed as n(%) or median(interquartile range) unless otherwise indicated; ^¥^Estimated by MDRD equation; ^€^Estimated by CKD-EPI creatinine equation; ^≠^Estimated by CKD-EPI cystatin C equation.

AKI, Acute Kidney Injury; CRRT, continuous renal replacement therapy; BUN, blood urea nitrogen; GFR, Glomerular Filtration Rate; NGAL, Neutrophil Gelatinase-Associated Lipocalin; CKD-EPI, Chronic Kidney Disease Epidemiology Collaboration.

A comparison of the time from AKI to CRRT initiation showed that survivors received CRRT earlier than non-survivors (9 hours vs. 26 hours, *P* < 0.001). However, there was no statistically significant difference in the duration of CRRT between the survivors and non-survivors (78 hours and 67 hours, respectively; *P* = 0.607).

### Optimal timing for CRRT initiation in septic shock patients with AKI

A comparison of risk factors for ICU mortality according to Cox proportional hazard analysis is shown in Table 3. In the unadjusted analysis, a lower BUN level (hazard ratio [HR], 0.991; 95% CI, 0.983–0.999; *P* = 0.024) and SCr level (HR, 0.768; 95% CI, 0.646–0.914; *P* = 0.003) at the time of AKI seemed to be related to ICU mortality, but this finding was not statistically significant in the adjusted analysis. A higher SOFA score (HR, 1.124; 95% CI, 1.044–1.211; *P* = 0.002), APACHE II score (HR, 1.036; 95% CI, 1.011–1.062; *P* = 0.005), and SAPS II score (HR, 1.028; 95% CI, 1.014–1.043; *P* < 0.001) at the time of ICU admission were found to related to increased ICU mortality, and the SOFA score was also significant in the adjusted analysis (HR, 1.106; 95% CI, 1.005–1.217; *P* = 0.038). The use of a mechanical ventilator (HR, 4.194; 95% CI, 1.908–9.221; *P* < 0.001) and the higher lactate level (HR, 1.239; 95% CI, 1.138–1.350; *P* < 0.001) at CRRT initiation were also found to be related to increased ICU mortality, but a low MAP (HR, 0.987; 95% CI, 0.977–0.998; *P* = 0.020) at ICU admission was statistically significant only in the unadjusted analysis. The longer the interval time from AKI to CRRT initiation, the higher the ICU mortality (HR, 1.016; 95% CI, 1.008–1.025; *P* < 0.001), but the duration of CRRT application was not a risk factor of ICU mortality.

**Table 3:** Cox proportional hazard analysis for ICU mortality.

	Unadjusted HR			Adjusted HR		
	HR	95% CI	*P* value	HR	95% CI	*P* value
Age	0.995	0.979–1.010	*0*.*505*			
Sex	1.126	0.722–1.758	*0*.*600*			
At acute kidney injury occurrence						
Lactate	1.055	1.013–1.100	*0*.*011*	0.862	0.793–0.937	<*0*.*001*
BUN	0.991	0.983–0.999	*0*.*024*			
Creatinine	0.768	0.646–0.914	*0*.*003*			
Potassium	0.826	0.662–1.031	*0*.*091*			
At ICU admission						
SOFA score	1.124	1.044–1.211	*0*.*002*	1.106	1.005–1.217	*0*.*038*
APACHE II score	1.036	1.011–1.062	*0*.*005*			
SAPS II score	1.028	1.014–1.043	<*0*.*001*			
Mechanical ventilator used	2.826	1.560–5.121	*0*.*001*	4.194	1.908–9.221	<*0*.*001*
Mean arterial pressure	0.987	0.977–0.998	*0*.*020*			
Procalcitonin	0.994	0.987–1.001	*0*.*112*			
At CRRT initiation						
Lactate	1.102	1.062–1.143	*<0*.*001*	1.239	1.138–1.350	*<0*.*001*
NGAL	1.000	1.000–1.000	*0*.*941*			
Cystatin C	0.993	0.832–1.184	*0*.*937*			
Interval time from AKI to CRRT initiation	1.005	1.002–1.009	*0*.*002*	1.016	1.008–1.025	<*0*.*001*
CRRT duration	*0*.*999*	0.998–1.001	*0*.*511*			

BUN, blood urea nitrogen; SOFA, Sequential Organ Failure Assessment; APACHE II, Acute Physiology and Chronic Health Evaluation Score II; SAPS II, Simplified Acute Physiology Score II; NGAL, Neutrophil Gelatinase-Associated Lipocalin; AKI, acute kidney injury; CRRT, continuous renal replacement therapy.

### Mortality rate of patients who initiated CRRT early and late

**Figure 1:**
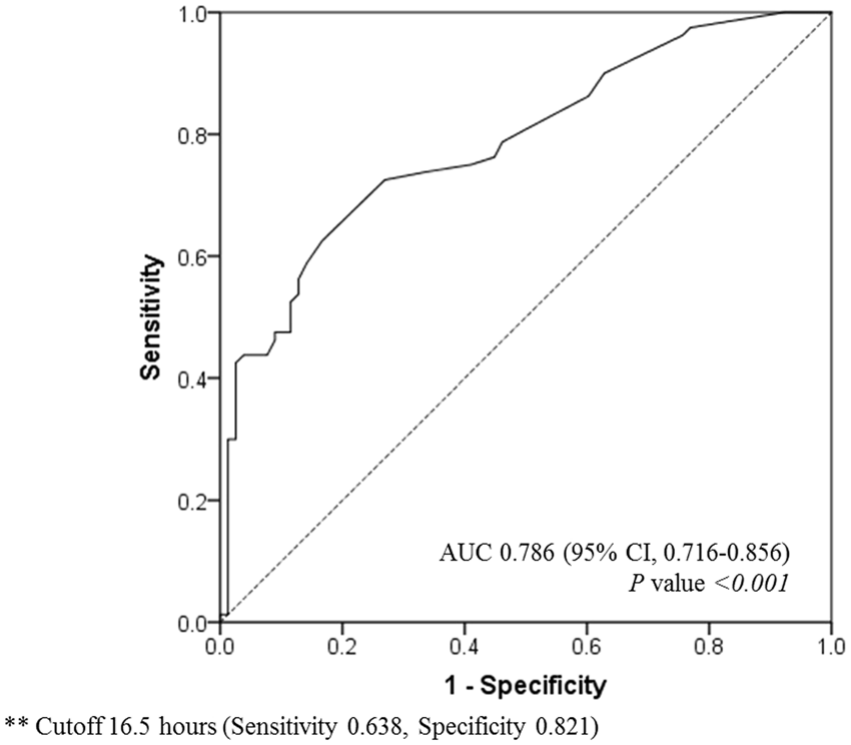
AUROC curve of optimal CRRT initiation time for ICU mortality. The cut-off value of the optimal interval time for ICU mortality.

The area under the receiver operating characteristic (AUROC) curve of the interval time from AKI to CRRT initiation for ICU mortality was 0.786 (95% CI, 0.716–0.856; *P* < 0.001). The cut-off value of the optimal interval time was within 16.5 hours (sensitivity 0.638, specificity 0.821), as shown in Fig. 1.

The overall mortalities at 28, 60, and 90 days of patients for whom CRRT was initiated within and after 16.5 hours were compared using the Kaplan-Meier curve (Fig. 3). The 28-day mortality rates were 70.8% (46/65 patients) in the group that initiated CRRT after 16.5 hours and more than 40.7% (38/93 patients) in the patients who initiated CRRT within 16.5 hours (log-rank test, *P* < 0.001; HR 2.118; 95% CI, 1.375–3.261). Cumulative mortality rates at 60 days (log-rank test, *P* < 0.001; HR 2.244; 95% CI, 1.497–3.363) and 90 days (log-rank test, *P* < 0.001; HR 2.115; 95% CI, 1.424–3.141) were also statistically significant in both two groups.

**Figure 2:**
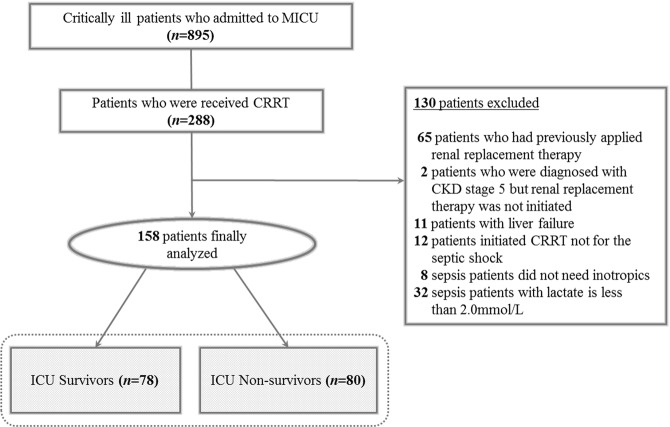
Study flow. Septic shock patients with AKI who was analyzed in the study.

**Figure 3:**
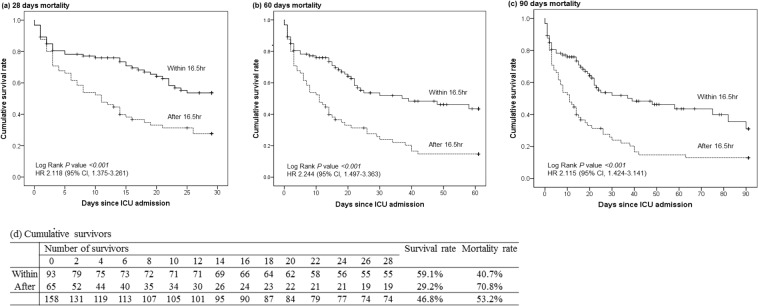
28 days-, 60 days- and 90 days- overall mortality of septic shock patients who initiated CRRT within and after 16.5 hours. The comparison of overall mortalities at 28, 60, and 90 days for early and late CRRT initiation groups. Within: time interval from AKI to CRRT initiation <16.5 hours; After: time interval from AKI to CRRT initiation ≥16.5 hours.

## Discussion

Renal failure is associated with high mortality in critically ill patients, especially patients with septic shock. Several trials have determined ways to predict decreased renal function and to improve clinical outcomes^16–18^. Recently, a new method, using machine leaning, is being studied to develop a prediction algorithm for AKI^19^. However, various clinical situations must be considered, and in some cases, renal dysfunction progresses rapidly, so it is still not easy to predict decreased renal function early. Thus, appropriate interventions at the right time are more important factors for improving hospital outcomes of septic shock patients with renal failure.

In critically ill patients, organ failures are associated with mortality, and the RRT in patients with AKI is known to improve the clinical course^20^. Especially, in patients with septic shock, RRT plays an additional role in modulating immune responses and influencing circulating levels of inflammatory mediators, unlike with other patients primarily requiring replacement of renal function^5,7^. And it is known to be more effective and hemodynamically stable in CRRT than IHD^6^.

However, in the KDIGO guideline, the initiation time of RRT is described to as ‘when life-threatening changes’, and it is more ambiguous when trying to initiate the CRRT clinically, because physicians are required to consider various situations^9^. Therefore, many researches on the timing and variables associated with the initiation of CRRT have been performed. In the prospective study, time limits for early and late initiation of CRRT were set up previously, and various outcomes including the survival rate were observed in the two randomized groups. However, because of the ethical concern associated with the time limit of the intervention, life threatening patients were excluded and time limits for initiation of CRRT was not enough long. Since patients with septic shock are at high severity and intervention within a time has an impact on survival rate, it is not an appropriate way^12,21^.

In this study, we retrospectively reviewed medical records and determined the time of AKI occurrence according to the KDIGO definition. Focused on the mortality, clinically most important outcome, we compared the interval time from AKI to CRRT initiation to find optimal time points without setting the time limit first. Even if patients were admitted to the ICU, the cases who had applied CRRT not for the septic shock were excluded from the study.

895 patients were admitted to the medical ICU about 2 years, 158 of whom were enrolled to our research. Our study population included severe septic shock patients who did not simply occurred AKI but also required CRRT. Their severity was demonstrated in Table 1 as SOFA, APACHE II and SAPS II score. Unexpectedly, the renal function at the time of AKI occurrence, WBC count, and procalcitonin level, which indicates the severity of infections, were higher in survivors, but the HR for ICU mortality did not increase statistically. However, the interval time from AKI to CRRT initiation and mechanical ventilator use were different between the groups. It took a longer time to initiate CRRT after AKI in non-survivors than in survivors, and non-survivors used mechanical ventilator more frequently than survivors. The HR was also proven to increase ICU mortality significantly, suggesting that the interval time from the onset of AKI to CRRT initiation is an independent risk factor for ICU mortality. Only the patients who did not agree with the do not resuscitate (DNR) order and who were assertive in treatment were enrolled; the duration of CRRT was long enough in both groups, and the patients had no significant risk factors.

The causes of infection were slightly different between the groups according to outcomes. Urinary tract infection was more frequent in survivors than in non-survivors, while respiratory infection was more common in non-survivors than survivor, although there was no statistical difference. We cautiously suggest that clinician’s follow-up and estimate the decrease in renal function more carefully in septic shock patients with urinary tract infection, and initiate CRRT earlier in these patients. CKD stage 5, which is an indication of RRT, was an exclusion criterion when selecting the study population, and there was no statistically difference in a history of CKD as a comorbid disease according to survival (Table 1).

In patients with septic shock, RRT is thought to improve the survival rate by correcting acidosis and inflammatory mediators^3,4^. However, there is still controversy regarding the modality of dialysis and optimal timing of initiating interventions for survival benefit^13,15,22^. On the basis of our study’s results, we think that CRRT initiation within 16.5 hours increases the survival rate in patients with septic shock. However, many of the previous prospective studies on ICU patients found that early CRRT did not increase the survival rate in these patients^10,11,23–26^. The conclusion that early CRRT is related with an increased survival rate was mostly proven by a retrospective study^13,14,27^. Perhaps, in the prospective study, the interval time for early versus late CRRT could not be long enough to make a difference in ICU mortality.

Most studies have shown that the appropriate initiation time of CRRT for survival benefit is usually within 24 hours form AKI, but the range varied from 8 hours to 18 days^10,12,28^. Herein, we analysed the interval time to predict ICU mortality using the receiver operating characteristic curve. The optimal timing for initiating CRRT was within 16.5 hours from AKI occurrence. There was also significant survival benefit in the group that initiated CRRT within 16.5 hours from AKI onset when comparing overall mortalities at 28, 60, and 90 days.

The severity of patients enrolled in this study was high because they were septic shock, and this is one of the strengths of this study compared with previous prospective studies^11,25^. This 16.5 hours is located in the gray zone, which is not included in the protocols of early *vs*. late application in recent two prospective studies [Artificial Kidney Initiation in Kidney Injury (AKIKI) trial^11^ (within 4 hours [vs.] until 72 hours) and Initiation of Dialysis Early Versus Delayed in the Intensive Care Unit (IDEAL-ICU] trial^25^ (within 12 hours [vs.] after 48 hours)]. Because of the property of a prospective study, patients with life-threatening conditions have to be excluded. Few studies have evaluated patients with septic shock; however, most of them included all patients admitted to the ICU, so the severity of patients was not high. Thus, it was difficult to apply these results directly to septic shock patients with a relatively high severity in clinical practice.

According to the KDIGO Clinical Practice Guideline for AKI, unlike the definition of AKI, the initiation time of RRT was not clearly defined; thus, it was based on clinical judgment. Therefore, the concept of distinguishing between early and late CRRT was defined arbitrarily depending on the authors, and time intervals from AKI to RRT was not long enough because of the ethical issue in prospective studies. Consequently, although early initiation of CRRT may affect the survival rate positively, it was difficult to suggest the appropriate timing for initiating CRRT. In the present retrospective study, we did not specify the concept of distinguishing early from late CRRT before the comparison, and the interval time of AKI to CRRT initiation was compared without the ethical concern associated with the time limit of the intervention. Therefore, we are convinced that the optimal timing of CRRT initiation suggested by our research findings is in accordance with the definition of early CRRT and could be generally applied in various clinical situations of septic shock patients. Furthermore, practical cut-off time from AKI to CRRT initiation is needed for the prospective validation study. In this regard, we presented the sensitivity, specificity, positive predictive value, negative predictive value, and accuracy for each cut-off time for ICU mortality in Supplementary Table 1. The Kaplan-Meier curve also showed a difference between the two groups even after 24 hours of cut-off. (Supplementary Fig. 1) Therefore, we expect future researchers to proceed with the prospective validation using cut-off time from 16 hours to 24 hours. But finally, practical cut-off time should be decided by each researcher in the future.

Nevertheless, this study has several limitations. First, RRT is known to improve the survival rate in septic shock patients with AKI, but it is necessary to compare whether CRRT or IHD is better. Because this study included septic shock patients with high severity who required ICU management, it is difficult to compare the detailed parameters of RRT, such as the dialysis interval, period of dialysis, and choice of CRRT or IHD. However, in patients who have suspected infections more frequently than septic shock, it is also necessary to determine which modality would be better considering the risks and benefits, clinically. Second, this study was conducted with a small sample size from a single institution, and it is not yet possible to generalise our findings to various patient groups. Future validation studies of other types of ICUs and multicentre studies are needed. Third, we could not analyse the specific reasons for CRRT initiation in septic shock patients with AKI. Subgroup analysis according to the reasons for CRRT initiation may have an impact on the prognosis of septic shock patients with AKI.

The timing of initiating CRRT cannot be determined based on only a few variables; thus, it is necessary to consider various clinical situations. Herein, we tried to determine the appropriate timing that could be applied to various clinical conditions, especially septic shock. We recommend careful observation when treating septic shock patients with AKI, and CRRT should be initiated within 16.5 hours of AKI onset, if possible, to help increase survival rates.

## Methods

### Study design and patient population

This study was a single-centre, retrospective cohort study, and the study protocol was approved by the institutional review board (IRB) of Severance Hospital (IRB number: 4–2018–0769) and performed in accordance to the Helsinki declaration. Individual patient data were stored as encrypted file and only authorized investigators were able to access the personal medical records to protect the privacy of patients. Informed consent was waived because the study was retrospective in design.

We analysed patients aged ≥18 years who were admitted to the medical ICU and received CRRT in the Yonsei University Severance Hospital from July 2016 to May 2018. Eight hundred ninety-five patients were admitted to the medical ICU, of whom 288 patients did not consent to the DNR order and received CRRT. One hundred thirty patients were excluded: those who were previously on dialysis (n = 65); those diagnosed as having chronic kidney disease (CKD) stage 5, which is an indication of RRT, but did not start dialysis (n = 2); those with liver failure in which the lactate level may have increased due to liver function deterioration (n = 11); those who received CRRT because of an electrolyte imbalance, volume control, uremic complications, and acid-base balance, not because of septic shock (n = 12); and those with sepsis without shock status (n = 40). Finally, 158 patients who initiated CRRT because of septic shock were enrolled, and the interval time from AKI to CRRT initiation according to ICU mortality was evaluated (Fig. 2).

### Variables and definitions

To determine the onset time of AKI, we reviewed patients’ medical records to determine the change of SCr and urine output measured before CRRT initiation. AKI was diagnosed based on the KDIGO Clinical Practice Guideline for AKI definitions. AKI was defined as any of the following: an increase in the SCr level by ≥0.3 mg/dL (≥26.5 µmol/L) within 48 hours; an increase in the SCr level to ≥1.5 times the baseline, which is known or presumed to have occurred within the last 7 days; or a decrease in the urine volume <0.5 mL/kg/h for 6 hours. If a patient presented with AKI without a baseline SCr level, it could be estimated using the Modification of Diet in Renal Disease (MDRD) Study equation, assuming that the baseline eGFR is 75 mL/min per 1.73 m^2^ = 186 × (SCr)–1.154 × (age)–0.203 × (0.742 for women) × (1.210 for black patients)^9^. As the renal function parameters, SCr and eGFR were calculated using the MDRD equation, Chronic Kidney Disease Epidemiology Collaboration (CKD-EPI) creatinine equation, and CKD-EPI Cystatin C equation at the initiation of CRRT; additionally, the neutrophil gelatinase-associated lipocalin and cystatin C levels were recorded.

When the urine output was recorded hourly, the time point of decreased urine volume <0.5 mL/kg/h for 6 hours was defined as the time of AKI occurrence. Otherwise, the time point assumed that the increased SCr level was ≥0.3 mg/dL or ≥1.5 times the baseline based on the laboratory findings. The values of other parameters were measured within 24 hours at the time of CRRT initiation and ICU admission.

Septic shock was defined based on The Third International Consensus Definitions for Sepsis and Septic Shock. Sepsis was diagnosed when the acute change of at least 2 points in the Sequential Organ Failure Assessment (SOFA) score was associated with suspected infection. Septic shock was a subset of sepsis, and the following criteria were required for diagnosis: a sepsis status and the requirement of vasopressor therapy to increase the mean arterial pressure (MAP) ≥65 mmHg and increase the lactate level >2 mmol/L (18 mg/dL) despite adequate fluid resuscitation^29^.

### Clinical outcomes

We performed the study by setting the study population and goals close to the aforementioned definitions and criteria as possible. First, based on the KDIGO guideline, which is accepted undisputedly, we determined the time of AKI occurrence, and then we calculated the time to initiation of CRRT.

As the primary outcome, the optimal interval time was analysed according to ICU mortality using the AUROC curve. After determining the cut-off time by the AUROC curve, we divided patients into the two groups who initiated CRRT within and after 16.5 hours without previously specify the point to distinguish between early versus late CRRT. The secondary outcome was a comparison of the overall mortalities at 28, 60, and 90 days between the two groups.

### Statistical analysis

The baseline demographics of the study patients were compared using descriptive statistics. Categorical variables are expressed as numbers with percentages, and continuous variables are presented as medians with interquartile ranges, which were analysed using the Mann-Whitney test. Cox proportional hazard analysis was performed to identify the variables associated with ICU mortality. Overall mortalities at 28, 60, and 90 days in patients with early versus late CRRT initiation were compared by Kaplan-Meier analysis.

A two-tailed 95% confidence interval (CI) was used, and a *P*-value ≤ 0.05 was regarded as significant. All statistical analyses were performed using SPSS Statistics, version 23 (IBM Corp., Armonk, NY, USA).

## Supplementary information

Supplementary table 1 and figure 1

## Data Availability

All data generated or analysed during this study are included in this published article.
