# Supplementary material for: Optimal timing of initiating continuous renal replacement therapy in septic shock patients with acute kidney injury
Source: Sci Rep. 2019 Aug 19;9:11981. doi: 10.1038/s41598-019-48418-4 (PMC6700095; doi:10.1038/s41598-019-48418-4)
Supplement: Supplementary file 1 — Supplementary table 1 and figure 1 [file 41598_2019_48418_MOESM1_ESM.docx]

| Cut-off time | Sensitivity (%) | Specificity (%) | Positive predictive value (%) | Negative predictive value (%) | Accuracy (%) |
| --- | --- | --- | --- | --- | --- |
| Within 6 hours | 96.3 | 24.4 | 56.6 | 86.4 | 60.8 |
| Within 12 hours | 73.8 | 66.6 | 69.4 | 71.2 | 70.3 |
| Within 16.5 hours | 63.8 | 82.1 | 78.5 | 68.8 | 72.8 |
| Within 18 hours | 58.8 | 85.9 | 81.0 | 67.0 | 72.2 |
| Within 24 hours | 52.5 | 88.5 | 82.4 | 64.5 | 70.3 |
| Within 48 hours | 33.8 | 97.4 | 93.1 | 58.9 | 65.2 |

**Supplementary Table 1. Comparison of each cut-off value for ICU mortality**

**Supplementary Figure 1. 28 days-, 60 days- and 90 days- overall mortality of septic shock patients who initiated CRRT within and after 24 hours**


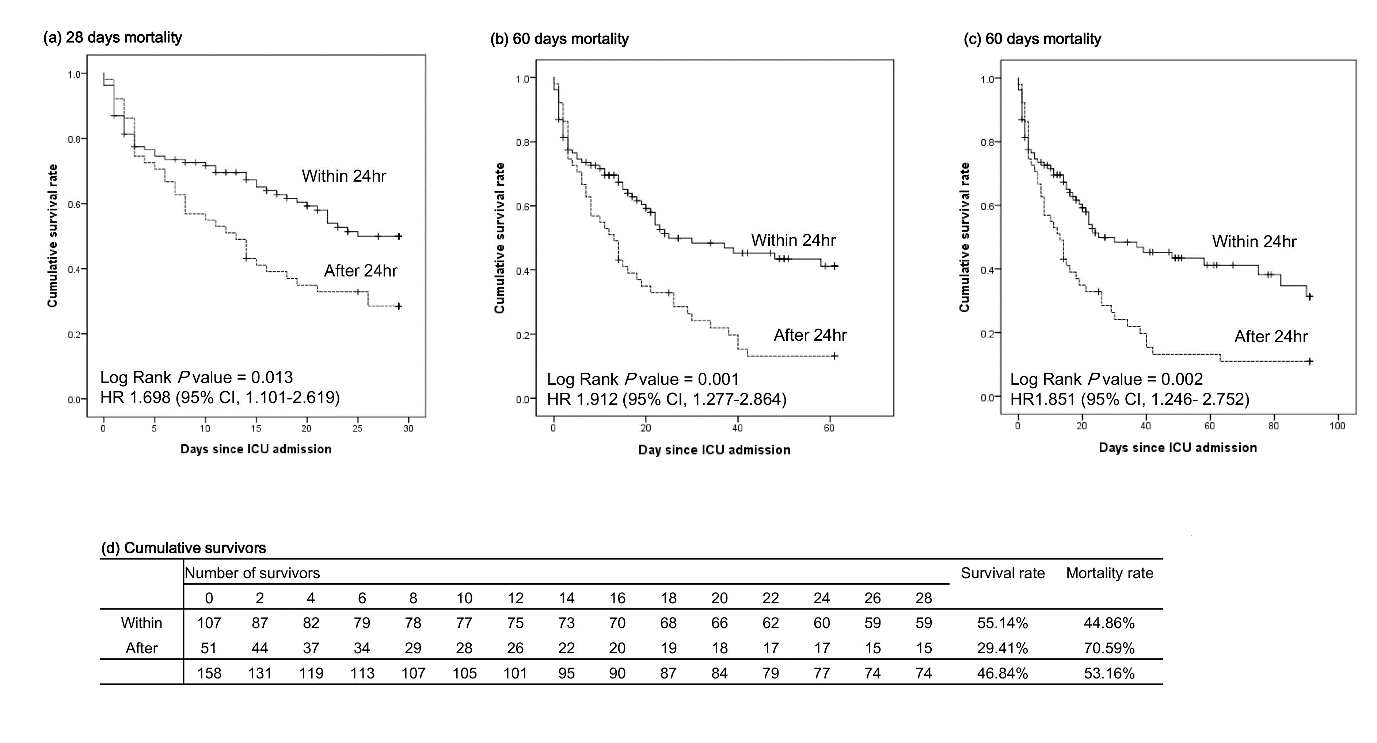


The comparison of overall mortalities at 28, 60, and 90 days for early and late CRRT initiation groups. Within: time interval from AKI to CRRT initiation<24hours; After: time interval from AKI to CRRT initiation≥24hours
